# Genetic Analysis by nuSSR Markers of Silver Birch (*Betula pendula* Roth) Populations in Their Southern European Distribution Range

**DOI:** 10.3389/fpls.2020.00310

**Published:** 2020-03-24

**Authors:** Giovanbattista D. de Dato, Angela Teani, Claudia Mattioni, Filippos Aravanopoulos, Evangelia V. Avramidou, Srdjan Stojnic, Ioannis Ganopoulos, Piero Belletti, Fulvio Ducci

**Affiliations:** ^1^Research Centre for Forestry and Wood, Council for Agricultural Research and Economics, Arezzo, Italy; ^2^Research Institute on Terrestrial Ecosystems, National Research Council, Porano, Italy; ^3^Faculty of Agriculture, Forestry and Natural Environment, Aristotle University of Thessaloniki, Thessaloniki, Greece; ^4^Institute of Lowland Forestry and Environment, University of Novi Sad, Novi Sad, Serbia; ^5^DI.S.A.F.A., Department of Plant Genetics and Breeding, University of Turin, Grugliasco, Italy

**Keywords:** marginal populations, *Betula pendula* (silver birch), population structure analysis, nuclear SSR markers, gene flow

## Abstract

In the main distribution area the genetic pattern of silver birch is dominated by two haplotypes: haplotype A located in the western and north-western Europe, and haplotype C in eastern and southeastern Europe, characterized by high levels of neutral genetic variability within populations, and low differentiation among populations. Information about the amount and structure of genetic variation in the southern marginal areas, representing rear populations left during the expansion of this species from southern glacial refugia, are lacking. The general aim of the study was to investigate the existence of the climatic characteristics typical of the environmental niche of the species, jointly to genetic organization, variation and gene flow, in marginal populations on the Italian Apennines and Greek Southern Rhodope and compare them with populations of the southern part of the main distribution range on the Alps and Balkans. Genetic analysis was performed using nuclear microsatellites loci on 311 trees sampled from 14 populations. Environmental analysis was performed on the multivariate analysis of derived climatic variables. The allelic pattern was analyzed to assess genetic diversity, population diversity and differentiation, population structure and gene flow. The geographic and environmental peripherality did not always match, with some Apennine sites at higher elevation enveloped in the environmental niche. In the peripheral populations on the Apennines, we observed a lower genetic diversity and higher differentiation, with evident genetic barriers detected around these sites. These characteristics were not shown in the marginal Greek populations. Unexpectedly, the southern Italian marginal populations showed genetic links with the Greek and central area of the distribution range. The Greek populations also showed evident gene flow with the Alpine and Balkan areas. The disparity of results in these two marginal areas show that it is not the geographic peripherality or even the ecological marginality that may shape the genetic diversity and structure of marginal populations, but primarily their position as part of the continuous range or as disjunct populations. This outcome suggests different considerations on how to manage their gene pools and the role that these rear populations can play in maintaining the biodiversity of this species.

## Introduction

Geographical distribution of European tree species and their genetic structure are the result of the climatic oscillations throughout the Quaternary (Hewitt, 2004). Many studies report that during the last glacial age temperate trees have been able to survive in areas of favorable environmental conditions in southernmost latitudes in Europe (Stewart et al., 2010), occupying the three southern peninsulas (Iberian, Apennine, Balkan). Concurrently, especially for boreal species, some refugia have also been found at higher latitudes, in central and eastern Europe, such as on the north of the Alps, Urals, or the Russian plain (Willis and van Andel, 2004; Svenning et al., 2008; Magri, 2010; Väliranta et al., 2011). Long-term persistence in several separated refugia during glacial episodes has been considered essential for population divergence (Hampe and Petit, 2005) since isolated intraspecific microevolution led to genetic differentiation among refugia (Harter et al., 2015). By the end of the glacial age (around 12,000 years BP), tree species started to migrate and expand from refugia. The genetic admixture of long-isolated lineages originating from different refugia created novel genetic variation in contact zones outside the glacial refugia (Petit et al., 2003; Havrdová et al., 2015). Also, some populations remained in the rear during the expansion. Nowadays, these rear populations constitute marginal and peripheral (MaP) populations (Hampe and Petit, 2005). Marginal populations, compared to the so-called “central (or core) populations,” are defined as those populations growing at the edges of their ecological niche space in ecologically and climatically marginal conditions, while peripheral populations are those growing at the edge of the geographic distribution range or that might be isolated from the continuum of the natural distribution. They might be poorly connected or not connected via gene flow with other populations (Fady et al., 2016).

Silver birch (*Betula pendula* Roth) is a diploid (2*n* = 28), monoecious, wind-pollinated tree with small seeds that rapidly recolonize open areas. Its current distribution range covers central Europe, the Balkans and northern Europe, where the distribution is nearly continuous in pure stands and mixed forests. In the western and southern parts of the range, as in the Italian Apennines and southern Rhodopes (northern Greece) the distribution is patchier and occurs mostly at higher altitudes (Vakkari, 2009). The main range is dominated by two haplotypes: haplotype A located in the western and north-western Europe, and haplotype C in eastern and southeastern Europe (Palmé et al., 2003; Maliouchenko et al., 2007; Tsuda et al., 2017), with an area of suture zone spread over northern Sweden, Poland, Hungary, and Romania. This picture suggests the absence of well-defined last glacial maximum (LGM) refugia. In fact, it is reported that birch was not only confined to southern European refugia, but was also spread in central and eastern Europe, specifically north of the Alps, near the Ural Mountains and in southern Sweden (Palmé et al., 2003; Normand et al., 2011; Jadwiszczak, 2012). The migration from these northern refugia resulted in the current presence of the two main dominant haplotypes. Migration waves from southern Italy at the end of the LGM were probably inhibited by the Alps, and did not contribute to the postglacial recolonization of birch in the rest of Europe at higher latitudes (Palmé et al., 2003; Birks and Willis, 2010; Jadwiszczak, 2012). On the other hand, southern Balkans Greek populations did not show any particular divergence compared to the central Balkans ones (Palmé et al., 2003). As a result, a clear genetic boundary can be pictured by distinguishing central-southern Europe from the rest of the range (Palmé et al., 2003). In the main distribution area, the genetic patterns of silver birch evidenced a high neutral genetic variability within populations, a low differentiation among populations, and a weak genetic structure, compared to most of the other broadleaved tree species (Palmé et al., 2003; Maliouchenko et al., 2007; Tsuda et al., 2017). Nevertheless, the southernmost birch populations might be poorly connected via gene flow with other populations and, as long-isolated, can encounter higher genetic differentiation and lower genetic diversity related to stochastic events such as chronic genetic drift, low gene flow and excess of inbreeding. However, MaP populations are usually exposed to harsh environmental conditions, which can act as an intense selective pressure for individuals; consequently, they may have an intrinsic evolutionary potential for adaptation and speciation (Sexton et al., 2009). In this context, filling the gap regarding the extent and the organization of genetic variation in the southern marginal areas can help to reduce the risk of loss of biodiversity by identifying populations with high values of genetic variability and merit the most attention in terms of conservation priority. Under the threats and challenges opened by climate changes, these populations can be a source of genetic novelty for reinforcing standing genetic variation in various parts of the range (Fady et al., 2016).

In this study, we investigated the genetic diversity and structure of birch populations focusing on the southern-central distribution range, particularly on the MaP populations on the Italian Apennines and Greek Southern Rhodope distributed at the boundary of the continuous range in scattered and rear locations. We included in the analysis populations from the Italian Alps and central Balkans, representing the southern part of the continuous distribution range, with climatic characteristics typical of the environmental niche of this species. We first tested if these peripheral populations represent sites at the margin of the environmental niche of the species, so that we could verify if peripherality could also affect the main genetic parameters by evaluating populations genetic diversity and structure, gene flow and divergence; hence, we were able to identify valuable areas/reservoirs of genetic diversity and eventually, we wondered whether some of these populations were worth to receive a genetic conservation status.

## Materials and Methods

### General Sites’ Description and Environmental Analysis

**FIGURE 1:**
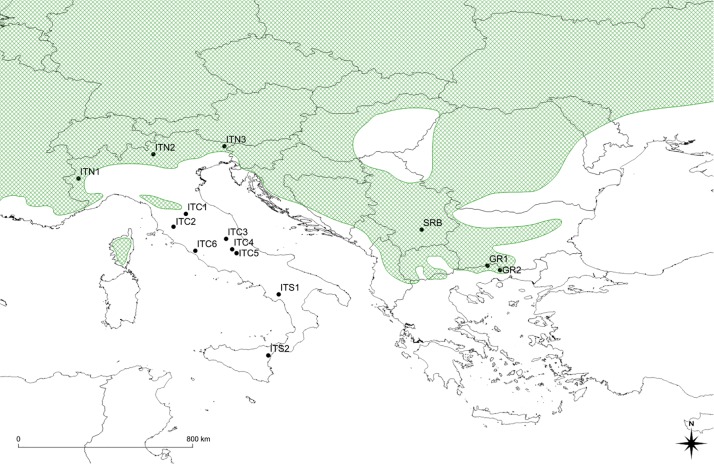
Map of sites’ location: black dots show position of sites; the green shaded area shows southern portion of the distribution range of *Betula pendula* (modified from Caudullo et al., 2017). ITC1, ITC3, ITC4, ITC5, ITS1, ITS2 cover small surfaces, with trees mainly scattered in broad-leaved mixed forests with beech (*Fagus sylvatica* L.) and chestnut (*Castanea sativa* Mill.). Populations extend to the altitudinal vegetation limit, on morainic sediments or loose soils, forming polycormic and short stands. ITC2 is on a peat bog at low elevation (460 m a.s.l.). It extends on only two small patches about 0.2 ha each, mainly with chestnut and *Alnus glutinosa* (L.) Gaertn. ITC6 is a pure birch stand around the remnant of an ancient crater, with sulfurous waters spring, at 260 m a.s.l. In the ITS2, birch is classified as *B. aetnensis* Rafin.; this is considered an endemic species of the Etna massif, typical of the volcanic soils, at altitudes between 1300 and 2100 m a.s.l. GR1 and GR2, silver birch populations co-exist with Scotch pine (*Pinus sylvestris* L.), European black pine (*P. nigra* Arn.) beech and sporadically Norway spruce [*Picea abies* (L.) H. Karst.]. The altitude varies around 1,000 m a.s.l. ITN1, ITN2, ITN3, and SRB represent core populations in the southern main distribution area.

The sampling sites covered a gradient from the Alps to Sicily and to Greece (Table 1 and Figure 1). Populations on the Apennines (ITC1, ITC2, ITC3, ITC4, ITC5, ITC6, ITS1, and ITS2) and southern Rhodopes in Greece (GR1 and GR2) are MaP populations disjunct from the main continuous distribution range. Populations on the Alps (ITN1, ITN2, and ITN3) and Balkans (SRB) represent populations in the southern main distribution area. In order to assess if geographical peripherality was related to environmental limitations, an ensemble of current climatic data was used to analyze the environmental variability of the study sites. The ClimateEU software^1^ was used. This software extracts and downscales Parameter-elevation Regressions on Independent Slopes Model (PRISM) monthly data (0.5° resolution) to scale-free point data (Daly et al., 2008) and calculates seasonal and annual climate variables for specific locations based on latitude, longitude, and elevation. The software was used to generate 15 biologically relevant climatic variables for the 1981–2010 normal period (Supplementary Table S1) and with 250 m of spatial resolution. A standardization procedure was applied as the variables are expressed in different scales. These variables were used to perform a principal component analysis (PCA). The PCA was performed in R (v3.6.1, R Core Team, 2019) by the default package STATS, in order to find variables (components) accounting for as much as possible of the variance in environmental data, avoiding collinearity between variables, an expected issue in ecological analysis.

**TABLE 1:** Sites locations.

Label	Site	Country		N°	E°	Altitude	N	Ar	Pa	Pa’	He	Fis
ITC1	Pratomagno	Italy	MaP	43.61	11.71	1070	22	5.55	0.62	5	0.74	−0.047
ITC2	Belagaio	Italy	MaP	43.08	11.20	460	20	6.35	0.53	4	0.71	0.0458
ITC3	Monti della Laga	Italy	MaP	42.58	13.37	1350	12	7.38	0.18	1	0.80	0.108
ITC4	Monte Sirente	Italy	MaP	42.15	13.62	1550	27	5.65	0.17	0	0.71	0.0004
ITC5	Monte Velino	Italy	MaP	41.99	13.80	1480	19	3.79	0.05	0	0.55	−0.072
ITC6	Caldara di Manziana	Italy	MaP	42.09	12.10	260	25	4.53	0.10	1	0.61	0.115*
ITS1	Cilento	Italy	MaP	40.29	15.54	1000	31	6.10	0.22	3	0.78	0.091
ITS2	Etna	Italy	MaP	37.77	15.11	1020	12	6.63	0.51	2	0.78	0.043
GR1	Simida Forest	Greece	MaP	41.47	24.16	1000	30	7.62	0.23	2	0.81	0.080
GR2	Erimanthos Forest	Greece	MaP	41.29	24.67	950	19	7.05	0.10	0	0.77	0.043
ITN1	Val Sangone	Italy	S-core	45.07	7.28	1400	18	7.87	0.35	2	0.82	0.050
ITN2	Val Camonica	Italy	S-core	46.07	10.37	920	30	7.63	0.36	3	0.80	0.063
ITN3	Val Raccolana	Italy	S-core	46.40	13.33	750	18	7.16	0.33	2	0.77	0.074
SRB	Ravna Planina	Serbia	S-core	42.96	21.44	990	28	7.07	0.34	4	0.77	0.026

Latitude and longitude in decimal degrees; altitude is the average altitude in m a.s.l. MaP, marginal and peripheral population; S-core, southern core population; N, total number of sampled individuals; AR, allelic richness; PA, number of private alleles with rarefaction; PA’, number of private alleles without rarefaction;HE, expected unbiased heterozygosity; FIS, fixation index for each sampled population. The symbol * indicates significant difference from the null value at *p* < 0.05.

### Plant Material and DNA Extraction

A total of 311 individuals were randomly sampled from the 14 populations (Table 1). Leaves or buds were collected from individual trees and put in silica gel for long-term conservation. Total genomic DNA was extracted by grinding 50–60 mg of dry material in liquid nitrogen using the DNeasy Plant mini kit (QIAGEN). Extracted DNA was quantified spectrophotometrically (Eppendorf Biophotometer^®^) and diluted to 5 ng/μl.

### Microsatellite Analysis

Samples were analyzed with a set of eight polymorphic markers (Table 2) developed by Kulju et al. (2004). PCRs were performed in triplex master mixes in Eppendorf Mastercycler^®^ pro. Each sample was amplified in a 12.5 μl total volume reaction according to the QIAGEN Type-it^®^ Microsatellite PCR kit. The cycling protocol included: 95°C for 5 min; 28 cycles at 95°C for 30 s, 57°C for 90 s, 72°C for 30 s; 60°C for 30 min. 1 μl of each amplification product was added in a mixture of 20 μl formamide (Applied Biosystems^TM^) and 0.3 μl GeneScan^TM^ 500 LIZ^TM^, and denatured at 95°C for 5 min. Samples were run on ABI Prism 3130 Avant DNA sequencer (Applied Biosystems^TM^). Results were analyzed using the GeneMapper^®^ Software v4.1 (Applied Biosystems^TM^), and allelic profiles were scored by automatic binning and visual checking.

**TABLE 2:** nuSSR markers used in this study.

Accession number	Locus	bp	Na	Ne	He	F	fna
AF310856	L1.10	168–209	30	6.0	0.82	0.002	0.021
AF310846	L2.2	132–155	11	2.7	0.60	−0.038	0.019
AF310851	L3.1	219–241	8	3.8	0.74	−0.010	0.016
AF310862	L5.4	230–262	24	5.0	0.77	0.032	0.031
AF310863	L5.5	121–146	27	7.5	0.88	0.172	0.062
AF310854	L7.1	146–152	7	2.9	0.66	−0.049	0.003
AF310864	L7.3	178–226	16	3.6	0.70	0.004	0.012
AF310866	L7.8	295–307	22	5.4	0.80	0.012	0.022

*Base pairs (bp), numbers of alleles (NA), effective numbers of alleles (NE), expected heterozygosity (HE) and fixation index (F), frequency of null allele (fna).*

### Statistical Analysis

#### Genetic Diversity, Population Diversity, and Differentiation

In order to assess the level of genetic diversity at the population level, measures of intra-population and inter-population genetic parameters were assessed by different approaches. The number of observed (NA), and effective (NE) alleles; observed (HO) and expected (HE) heterozygosity (Nei, 1973), were calculated using GENEALEX v6.5 (Peakall and Smouse, 2006, 2012). The inbreeding coefficient (FIS) for each population and the fixation index (F) at each locus were obtained by computing a hierarchical AMOVA using ARLEQUIN v3.5.2.2 (Excoffier and Lischer, 2010) and its statistical significance was determined by a non-parametric approach using 1,000 permutations. As the assessment of allelic richness by the measure of allele frequencies needs to consider the variation in population sizes, allelic richness (AR) and the number of private alleles (PA) were computed using the rarefaction method with HP-RARE software (Kalinowski,2005b). Null allele frequency (fna) for each locus was estimated by FreeNA (Chapuis and Estoup, 2007). Population differentiation (FST) was estimated in GENEALEX v6.5 (Peakall and Smouse, 2006, 2012). To examine if the number of sampling and markers were adequate to provide a reasonably picture of the genetic population structure, the program POWSIM (v4.1; Ryman and Palm, 2006) was used. POWSIM simulates populations that diverge from a common base population to a predefined true FST (as resulted from the AMOVA); each set of populations is then sampled, a test for genetic homogeneity is applied, and the proportion of significant outcomes is used to estimate the power (the chance of detecting the minimum amount of genetic differentiation) under the actual conditions. We set the program with an effective population size Ne = 150, number of generation *t* = 35 and 500 runs. To delineate the major ordination pattern of birch populations based on a multivariate approach, a discriminant analysis of principal components (DAPC) was performed by R (v3.6.1, R Core Team, 2019) using the package ADAGENET (v2.1.1) in the R Studio environment (v1.1.463, R Studio Team, 2016). The method transforms the genetic data into principal components and then uses k-means clustering to define groups of individuals, so that within-group variation is minimized, while among-group variation is maximized. We kept axes for PCA that explained about 90% of the variation and *k* = 8 was selected based on the distribution of the Bayesian Information Criterion (BIC). To test if any isolation by distance (IBD) has been occurred, correlation between genetic distance and geographic distance was tested in GENEALEX v6.5 by the Mantel test, with 999 permutations.

#### Detection of Bottleneck and Effective Population Size

We quantified drift by estimating the effective sizes of the studied populations (NE). We applied the three single-sample methods, namely the linkage disequilibrium (LD), heterozygote-excess (HE) and molecular coancestry (MC), as implemented in the software NEESTIMATOR v2.1 (Do et al., 2014). These three methods are different from temporal methods and do not require distinct samplings of the same population spaced over time to detect the effective population size. Since all methods have flaws and restrictions (for details see Nomura, 2008; Waples and Do, 2008; Zhdanova and Pudovkin, 2008; Do et al., 2014), the application of multiple methods to the same dataset in NEESTIMATOR allows obtaining a reliable interpretation of the results.

To detect evidence of recent bottlenecks in these *B. pendula* populations, we compared actual and expected gene diversity calculated from the observed number of alleles under the mutation–drift equilibrium hypothesis, by the BOTTLENECK software (Piry et al., 1999). When a significant number of loci shows excessive gene diversity, then the population is likely to have undergone a recent bottleneck (Luikart and Cornuet, 1998). The probability distribution was estimated using a Wilcoxon test with 10,000 simulations. For microsatellites, a two-phase model (TPM) was applied, following recommendation reported in Piry et al. (1999), setting single-step mutations at 95% and multi-step mutations at 5% and a variance among multiple steps of 12.

#### Population Structure

In order to define the number of gene pools in the dataset, we used different approaches to identify genetic clusters and barriers to gene flow, specifically Bayesian clustering algorithms, edge detection, and network analysis methods. Bayesian clustering without spatial information was applied by STRUCTURE v2.3.4 (Pritchard et al., 2000). Twenty iterations were run with a burning period of 10000 and 10^5^ Markov chain Monte Carlo cycles (MCMC). The most likely number of clusters was determined by the Evanno method (Evanno et al., 2005), using the STRUCTURE HARVESTER (Earl and vonHoldt, 2012). The runs were averaged by CLUMPP v1.1.2 (Jakobsson and Rosenberg, 2007) and graphically represented using DISTRUCT (Rosenberg, 2004).

For edge detection, we used the Monmonier’s algorithm, that detects the path through the strongest genetic distances between neighbors, identifying boundaries between populations where changes in allele frequencies occurred. For this purpose, we used the R-package ADAGENET (v2.1.1) in R (version 3.6.1, R Core Team, 2019) under the R Studio environment (v1.1.463, R Studio Team, 2016). A Euclidean distance matrix was defined for the 14 populations. The algorithm determines where genetic boundaries exist based on estimating the locations of the maximum pairwise distances.

To visualize and analyze the genetic relationships without assuming an *a priori* cluster of populations, we applied graph-based network theory analysis to create a minimum spanning network (MSN) of the genetic relationship of populations, implemented in EDENetworks v2.18 (Kivelä et al., 2015). The software plots populations as nodes in a network graph with connections between nodes weighted by their pairwise genetic distance (FST). EDENetworks illustrates the distribution of links among populations using percolation theory and without an *a priori* hypothesis based on population identity or sampling location. The percolation theory allows for the splitting of a fully connected network into discrete clusters, and the critical threshold distance is known as the percolation threshold (Kivelä et al., 2015).

## Results

### Environmental Analysis

**FIGURE 2:**
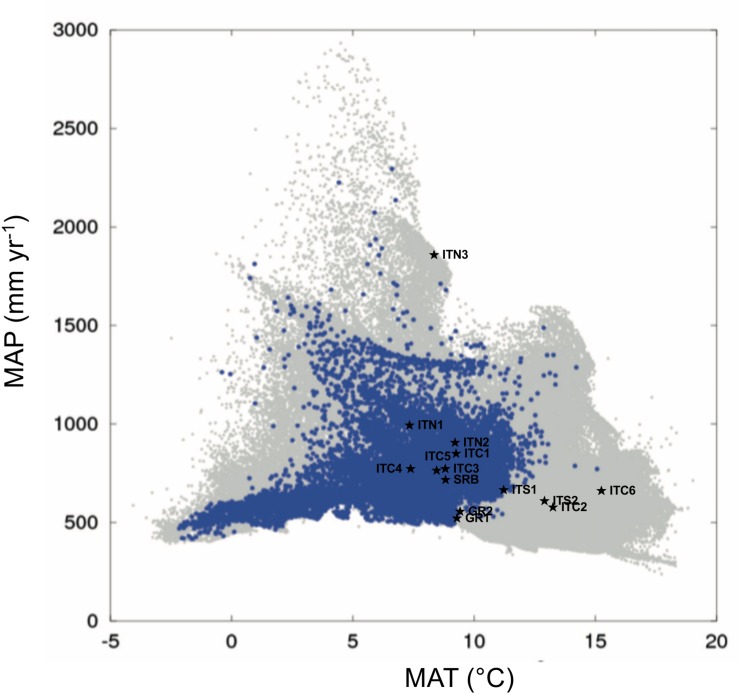
Autoecology diagram based on harmonized field observations from forest plots for *Betula pendula* (modified from Beck et al., 2016). MAP (mean annual precipitation) and MAT (mean annual temperature) of the sites of this study are those deriving by the ClimateEU as described in “Materials and Methods” section. The blue dots illustrate the specific climate niche of the *Betula* species. The overall climate space occupied by each of the field observations on all species included in the work by Beck et al. (2016) is represented by a gray spot. The symbols ★ show the study sites.

The PCA of the study sites (Supplementary Figure S2), based on the multivariate analysis of the 15 climatic variables derived in ClimateEU (Supplementary Table S1), separated sites based on their thermal demand along the first component (explaining 83% of the variance), and the precipitation and moisture deficit along the second component (explaining 13.7% of the variance). The autoecology diagram (Figure 2), based on the picture in Beck et al. (2016), showed that ITC6, ITC2, and ITS2 fell outside the climate niche of the *Betula* species; consequently, they can be considered ecologically marginal. ITS1 and the two Greek sites GR1 and GR2 are at the limit of the climatic niche. ITN3 is shown as an outlier, especially in term of precipitation, but without any limitation in terms of water and thermal demand. The other sites, that include SRB, the Alpine sites ITN1 and ITN2, and the Apennine sites ITC1, ITC3, ITC4, ITC5 can be considered within the envelope of the environmental niche.

### Genetic Diversity

A total of 145 alleles was observed across the eight loci (Table 2). The number of alleles per locus varied from 7 (L7.1) to 30 (L1.10), with an average of 18.1. The frequency of null alleles (fna; Table 2) was low, with an average of 2.3% over all loci. The fixation index (F; Table 2) ranged from −0.05 to 0.17, without any significant deviation from zero.

### Population Diversity and Differentiation

Allelic richness (AR) varied between 3.8 and 7.9 (Table 1). The lowest AR was detected in ITC5 and ITC6, while ITC3, ITN1, ITN2, ITN3, SRB, GR1, and GR2 showed an AR greater than 7.0.

On average, Italian MaP populations showed a lower level of AR compared to Greek MaP populations (5.7 and 7.3, respectively) and to the core populations ITN1, ITN2, ITN3, and SRB (7.4, *p* < 0.05). The three Balkan populations did not differ in terms of AR (on average 7.2).

The mean number of private alleles (PA; Table 1) ranged between 0.05 in ITC5 and 0.73 in ITC1. Italian Apennine sites showed an average PA equal to 0.35, but this value decreased along the North Apennines - Central Apennines gradient from ITC1 to ITC5 and then it rose again in ITS1 and ITS2. A low value of PA was recorded in Greek populations (on average 0.18). Southern core populations ITN1, ITN2, ITN3, and SRB showed a mean number of PA equal to 0.31.

The level of heterozygosity (HE; Table 1) was on average 0.71. Values ranged from 0.55 (ITC5) to 0.82 (ITN1). Among the Italian MaP populations, a mean HE equal to 0.71 was detected; ITC3 had the highest HE (0.80), while ITC6 and ITC5 presented the lowest values (0.61 and 0.55, respectively). Greek MaP populations (GR1 and GR2) tended to show a higher HE, compared to Italian MaP populations (0.79 vs. 0.71); In addition, their HE was not significantly different from that of the Alpine and SRB populations (both averaged 0.79).

The inbreeding coefficient (FIS; Table 1) ranged between −0.072 (ITC5) and 0.115 (ITC6). FIS for the Italian MaP populations was on average 0.036. A significant departure from FIS = 0 was measured in ITC6. In Greek MaP populations, FIS averaged 0.062. In the Alpine and Serbian populations, FIS was 0.062 and 0.026, respectively.

The power analysis by POWSIM, applied to the 14 populations sampled in this study, with an Fst = 0.11 and 8 SSR markers, resulted in a significant power of 1. When the drift steps were omitted (*t* = 0), the probability of error when assuming divergence among populations was α = 0.04.

**FIGURE 3:**
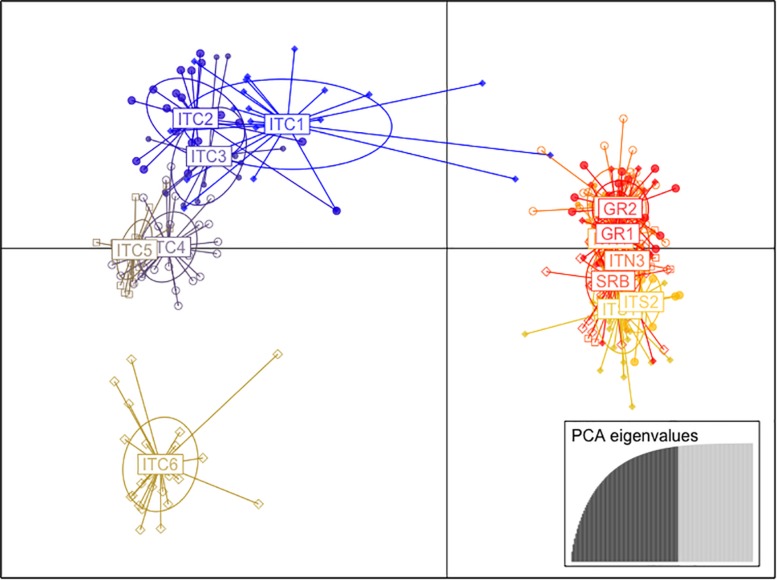
Scatter plot of the first and second principal coordinates based on the discriminant analysis of principal components (DAPC) of nuSSR markers.

The cluster analysis (DAPC; Figure 3) showed two main clusters formed along the first two coordinates. The first axis grouped the Italian populations of the central Apennines (ITC1, ITC2, ITC3, ITC4, ITC5, and ITC6), while the other embraced the two southernmost Italian sites (ITS1 and ITS2), the Greek populations (GR1and GR2), the Alpine sites (ITN1, ITN2, and ITN3) and the Balkans (SRB). There was further separation along the second axis, especially ITC6 was depicted as clearly isolated from both clusters.

Based on this evident existence of two main clusters, the analysis of molecular variance (AMOVA; Table 3) was performed separately. The cluster ITC1 – ITC2 – ITC3 – ITC4 – ITC5 – ITC6 (Table 3A) showed a highly significant level of differentiation among populations (FST = 0.146, *p* < 0.05), while diversity within populations (FIS = 0.022) resulted not significant. The other cluster ITS1 – ITS2 – ITN1 – ITN2 – ITN3 – SRB – GR1 – GR2 (Table 3B) presented comparable low values of FIS and FST (0.062 and 0.043, respectively).

**TABLE 3:** Analysis of molecular variance (AMOVA) for the two clusters as resulted from the DAPC and the STRUCTURE analysis: **(A)** for the cluster ITC1 – ITC2 – ITC3 – ITC4 – ITC5 – ITC6, **(B)** for the cluster ITS1 – ITS2 – ITN1 – ITN2 – ITN3 – SRB – GR1 – GR2.

	d.f.	SS	Variance components	%	F-statistic		p
**(A)**							
Among populations	5	110.634	0.469	15	FST	0.146	*
Within populations	119	332.518	0.061	2	FIS	0.022	ns
Within individuals	125	334.000	2.672	83	FIT	0.166	*
TOT	249	777.152	3.202	100			
**(B)**							
Among populations	7	69.551	0.143	4	FST	0.043	*
Within populations	178	599.839	0.196	6	FIS	0.062	*
Within individuals	186	554.000	2.978	90	FIT	0.102	*
TOT	371	1223.390	3.317	100			

d.f., degrees of freedom; SS, sum of squares; p, level of significance (* *p* < 0.005).

The FST pairwise population comparison (Table 4) revealed significant divergence among MaP Italian populations, presenting a mean FST equal to 0.15; on the contrary, GR1 and GR2 were not distinctively differentiated, a result extended to the comparison with SRB. The MaP Italian populations as a whole had an average differentiation when compared to the Alpine populations (ITC1, ITC2, and ITC3) of 0.11.

**TABLE 4:** FST pairwise comparison among populations.

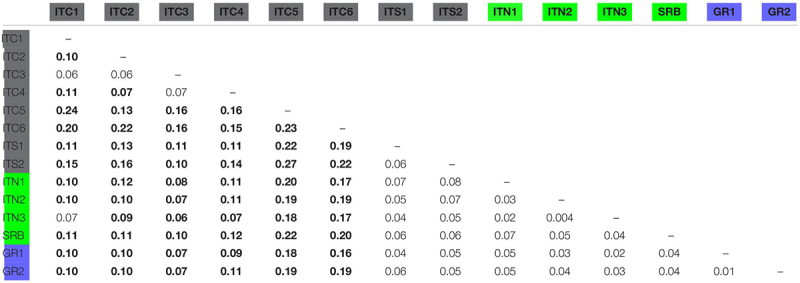

*Bold values are significant different from 0 at *p* < 0.05. Cell colors indicate Italian marginal populations in gray; core populations in the southern European range (in green); Greek marginal populations (in light blue).*

Considering all the Italian populations (the Alps to Sicily transect), the Mantel test resulted in no correlation between geographic and genetic distances (*r* = −0.27, *p* = 0.15). On the contrary, the transect along the Balkans, from northern Greece to SRB, throughout the Alps showed a weak positive significant correlation between geographic and genetic distances (*r* = 0.38, *p* < 0.05).

### Detection of Bottleneck and Effective Population Size

Effective population size (NE) showed large values, with confidence intervals (CI) ranging to infinite in most cases and for all methods (Table 5), without any evidence of genetic drift. The models used to detect population bottlenecks did not show any significant heterozygosity excess averaged across loci in all populations, thus proving the lack of any recent bottleneck event (Table 5).

**TABLE 5:** Estimation of effective population size (NE) estimated in NEESTIMATOR under the three different models: linkage disequilibrium (LD), heterozygosity excess (HE) and molecular coancestry (MC); probabilities from Wilcoxon signed-rank tests testing for heterozygosity excess, and mode shift, using Bottleneck, for the 14 birch populations (values in parentheses indicate 95% confidence intervals).

Label	Ne			TPM	Mode shift
	LD	He	MC		
ITC1	107.1 (25–∞)	∞ (6.2–∞)	64 (0.1–321)	0.156	Normal
ITC2	64.5 (24.6–∞)	∞ (37.1–∞)	22.6 (1.7–70.3)	0.963	Normal
ITC3	∞ (19.2–∞)	∞∞	∞∞	0.273	Normal
ITC4	36 (19.6–103	∞ (11.9–∞)	∞∞	0.473	Normal
ITC5	∞ (45.9–∞)	∞ (5.5–∞)	14 (1–43.6)	0.344	Normal
ITC6	140 (28–∞)	∞∞	15.5 (1.9–43.1)	0.473	Normal
ITS1	176.9 (48.9–∞)	∞∞	76 (0.1–381.5)	0.473	Normal
ITS2	∞ (27.3–∞)	∞ (16–∞)	∞∞	0.191	Normal
ITN1	∞ (79.1–∞)	∞ (28.9–∞)	∞∞	0.422	Normal
ITN2	162.9 (53.3–∞)	∞∞	∞∞	0.770	Normal
ITN3	62.1 (23.8–∞)	∞∞	10.2 (6.1–15.4)	0.527	Normal
SRB	47.2 (25.7–147.3	∞ (26.1–∞)	8.5 (3.3–16.2)	0.963	Normal
GR1	∞ (102.5–∞)	∞∞	18.5 (3.8–44.6)	0.770	Normal
GR2	∞ (72.6–∞)	∞ (26.7–∞)	∞∞	0.320	Normal

### Population Structure, Gene Flow, and Barriers

**FIGURE 4:**
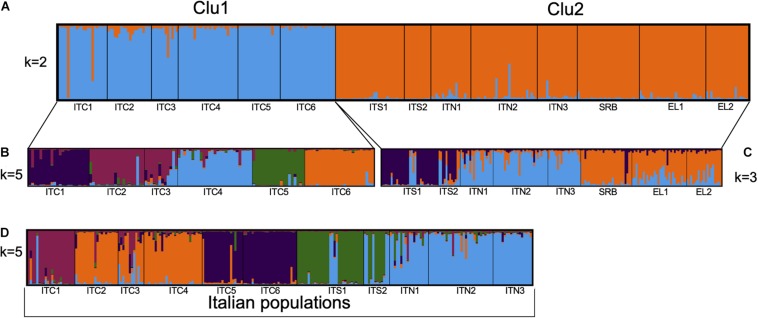
Estimated population structure obtained by STRUCTURE analysis based on eight nuSSRs: **(A)** for 311 individuals in the 14 populations for the cluster *k* = 2; **(B)** for 125 individuals in the six populations of central Apennines for the cluster *k* = 5; **(C)** for 186 individuals in the eight populations of southern Apennines, Alps, and Balkans for the cluster *k* = 3; **(D)** for 234 individuals in the 11 populations along the Italian peninsula for cluster *k* = 5.

The STRUCTURE analysis (Figure 4A and Supplementary Figure S1) revealed a highest likelihood partitioning at *k* = 2 which distinguished between one group that assembled all the Italian northern-central Apennines populations (ITC1, ITC2, ITC3, ITC4, ITC5, and ITC6; referred to as Clu1) and the other that included the southernmost Italian populations, the Alpine and the Balkan populations (ITS1, ITS2, ITN1, ITN2, ITN3, SRB, GR1, and GR2, referred to as Clu2).

Further analysis of the substructure of these two gene pools was performed. Clu1 presented *k* = 5 as the most likely group partitioning (Figure 4B). The substructure of Clu2 revealed *k* = 3 as the most likely number of genetic groups, in particular, the ITS1 – ITS2 group, the Alpine ITN1 – ITN2 – ITN3 group and the Balkan SRB – GR1 – GR2 one (Figure 4C). However, some admixture was evident in these groups. When we included only the Italian populations in the structure analysis, a separation into five groups (*k* = 5) was observed, with the Alpine populations sharing a common gene pool and the Apennines populations more differentiated among each other (Figure 4D).

**FIGURE 5:**
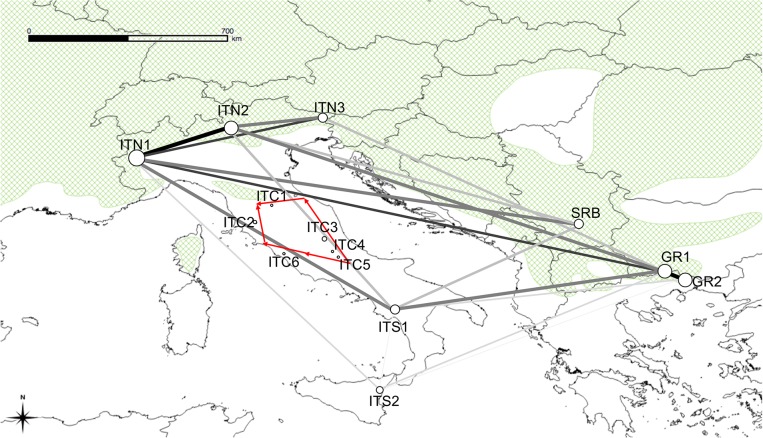
Map overlaid with network lineages identified by EDENetworks between nodes (sampling sites). Line thickness and colors are proportional to linkage strength and node size is proportional to the number of linkages for each node. The red arrows indicate the position of the barriers to gene flow identified by the Monmonier’s function implemented in ADEGENET.

The Monmonier’s function in ADEGENET recognized genetic barriers around the northern and central Apennine sites (Figure 5). The MSN resulted from EDENnetworks (Figure 5) displayed in the site ITN1 the main node (eight connections), followed by the two Greek sites and SRB (7), ITS1and ITN2 (6), ITS2 (5), and ITN3 (4). Low connections (from 0 to 1) were shown for the nodes in central Apennines populations. Thus, the resulted network (Figure 5) showed strong links among the Alpine sites with the Balkan ones and also, despite the physical distance, among the Alpine sites and the southern Italian sites. Medium strength links were displayed between Southern Italy and the Balkan peninsula. Poor or null connections existed among the northern and central sites in Italy.

## Discussion

In this study, we investigated the environmental variability and genetic structure of silver birch populations, covering the southern distribution range of the species. In particular, our aim was to explore how diversity and genetic structure was arranged in MaP populations, and compared them with populations from the southern part of the continuous distribution range. The sampling at the low latitudes of this widely distributed species, whose distribution is mainly centered at northern latitudes, can have an impact to understand genetic reservoirs of these MaP populations, whose preservation is important under the challenges deriving from climate changes.

### Environmental Analysis

Our distinction among the MaP populations with the central populations relied on geographic elements, assuming their distance and peripheral location reflect their ecological requirements, with environmental conditions being optimal at the center and poorer at the periphery (Brown, 1984). The environmental analysis performed in this study partially reflects this view. In fact, all the populations on the Alps and SRB fitted within the environmental niche depicted in the chorological map by Beck et al. (2016), based on datasets of field observations. ITN3 appeared as an outlier similarly to other scattered sites with high precipitation and cold temperatures. The southernmost MaP (ITS2) and the two MaP populations at the lowest elevation (ITC2 and ITC6) were characterized by environmental requirements outside the environmental niche for this species. These three sites all presented dry conditions due to high evaporative demand and elevated temperatures, conditions not typical of birch sites. Also, it is interesting to note that these last three mentioned sites (ITS2, ITC2, and ITC6) are peculiar in their soil substrates. The Greek sites (GR1 and GR2) together with ITC1, are sites located at the border toward dry and warm conditions similarly to ITS2, ITC2, and ITC6. If the future Mediterranean climate is drier and warmer (IPCC, 2013), these populations might face harsher climatic conditions. The other MaP populations of the Apennines (ITC1, ITC3, ITC4, and ITC5), all vegetating at high altitudes, are within the environmental niche, similarly to the Alpine and Serbian populations. Based on this ordination, our classification solely based on geographical elements is not biased, especially for the more elevated sites of the Italian Apennines. This basic view, when integrated with genetics, has to deal with the fact that MaP populations are usually smaller, numerically less abundant and more fragmented when compared to central populations; consequently the reduced effective population size and low gene flow can determine higher genetic differentiation and lower genetic diversity, as a result of stochastic events (genetic drift, low gene flow and excess of inbreeding), as it was the case for the Apennine populations, discussed below.

### Genetic Diversity

Our data displayed a high level of genetic diversity at the SSR loci examined in this study, with no sign of excess of inbreeding. Only locus L5.5 tended to show a high FIS, but this resulted not significant, with no alteration of the allelic frequencies from equilibrium. At the population level, the sites on the Italian Apennines (ITC1–ITC2–ITC3–ITC4–ITC5–ITC6–ITS1–ITS2) generally presented a lower level of allelic richness and, to less extent, gene diversity compared to the putative central populations ITN1, ITN2, ITN3, and SRB. Differently from the Italian MaP populations, Greek MaP populations revealed a higher allelic richness and heterozygosity compared to the Italian ones and similar to the Alpine and Serbian populations. This pattern highlights that stochastic events, like genetic drift and low gene flow, might have affected the genetic diversity of the Italian MaP populations, while displays the overall favorable conditions of the Greek populations, in terms of genetic diversity. GR1 and GR2 reflect the other accepted hypothesis about the MaP populations and their level of gene diversity. In fact, according to other authors, marginal populations may harbor the bulk of genetic diversity at the species level (Hewitt, 2004; Hampe and Petit, 2005), as range-wide patterns of genetic diversity are typically shaped by past climate-driven range dynamics than by stochastic events (Eckert et al., 2008). Hence, refugial Greek populations might have been source of migrants during the northward advancement of birch at the end of the last LGM, contributing to the colonization of Balkans and mixing with other central European refugial populations or migrants from the east waves (Petit et al., 2003; Palmé et al., 2003; Birks and Willis, 2010).

Different level of private allelic richness was observed among the Italian MaP populations, with the highest values in ITC1 and ITC2. A lower level of genetic diversity was observed in the southern part of the central Apennines (ITC5) and in ITC6, concomitant with a fewer number of private alleles and allelic richness, while the highest genetic diversity was observed in ITC4, ITS1, and ITS2. The detection of a high level of private alleles, as observed in ITC1, may help to detect putative refugial region; newly colonized areas are characterized by low genetic diversity and lack of private haplotypes, as might be the case for ITC5. However, according to Magri et al. (2015), multiple refugia made up by small isolated scattered populations had been present in these mountains, without any substantial change in extension and distribution. This can also be in accordance with the presence of multiple glaciers (Allen et al., 2008; Adamson et al., 2013) from the northern Apennines to Etna, indicating different spots of cold areas, thus presumably suitable habitats for birch. The ITC6 population, which presented low levels of allelic richness and private alleles, as well as a positive significant inbreeding coefficient, can have been affected by inbreeding among genetically related trees, likely due to selection pressure linked to the characteristic unfavorable environment of this site (low elevation and semi-arid climate, sulfuric water and acid soil). The structure and sub-structure analyses stressed the peculiarity of the site, confirming its genetic divergence. The physiological basis of the population survival under these conditions has not been investigated according to the authors’ knowledge. It is noted that birch fossil pollen has been found in the general area (at 15–24 ka BP), in a site along the Tyrrhenian central coast, inferring a long-standing presence of this species (Tzedakis et al., 2013). Genic selection under a long-lasting environmental pressure has likely led to a unique genetic composition and differentiation in this population. Genome sequencing of natural birch populations revealed that an evident link exists between environmental variable, meanly temperature and precipitation, and candidate gene expression related to cold acclimation and phenological events like cambial activity, wood formation, bud burst, and flowering (Salojärvi et al., 2017). Such genetic change induced by environmental pressure was manifested, for instance, in an intact grassland ecosystem in response to 15 years of simulated climate change (Ravenscroft et al., 2015). It is likely that this phenomenon would happen also in trees, but at different time scales (Kremer et al., 2014; Schierenbeck, 2017).

A slight gradient of diversity along the Apennines at the higher elevation was observed, with a high genetic diversity in the southern Italian MaP populations (ITS1 and ITS2), a lower value in ITC5 and a rise again toward ITC3 and ITC1. In a study on birch genetics by Tsuda et al. (2017), assessed by nuclear SSRs, that sampled Italian populations matching our sites ITN2, ITC1, and ITS1, and that also included a northern Greek population, a similar pattern of gene diversity and allelic richness can be observed, with the northern Apennine population showing a lower level of allelic richness and gene diversity compared to the Alpine, the southern Italian and Greek populations.

### Population Structure and Gene Flow

Two genetically different groups were distinguished, separating the six MaP populations in central Italy from the two southern Italian populations, which share their gene pool with Alpine, Serbian, and Greek populations. The separation of the six populations of the central Apennines from those in the southern Apennines resembles the result found for silver fir by Piotti et al. (2017). The uniqueness of the northern-central Apennines populations is strengthened by the genetic barriers detected in our Monmonier’s function analysis, showing the lack of gene flow along the Italian peninsula. As some sites are only a few kilometers apart, a mountain island effect and different environmental conditions could have provoked this phenomenon (Schoettle et al., 2012; Yousefzadeh et al., 2016), causing non-overlapping flowering phenology and shaping the pattern of genetic structure even in a species with potentially high gene flow (Ortego et al., 2012). The high statistical significance, resulting when analyzing the power of this structure, made us confident that the sample and marker sizes were sufficient to provide with high probability the depicted genetic population structure. It is often claimed that statistical power is expected to be high when sample and loci sizes are large. These conditions are particularly true at a low level of differentiation and short divergence time (Kalinowski,2005a). The level of Fst = 0.11 detected among these populations can be considered large enough to be caught through our sampling strategy, further supported by the high level of polymorphic loci.

In our study, geographic distances were not correlated to the genetic distances when the Italian populations were tested, which is typical when gene flow between neighboring populations is low, causing differentiation and weakening of the isolation-by-distance effect on the spatial genetic structure. The same result was found for European beech (*Fagus sylvatica* L.) populations in different European regions (Leonardi et al., 2012). They indicated habitat fragmentation and small population size as the causes of the genetic differentiation pattern observed in marginal beech populations. Although we did not detect any recent bottleneck or genetic drift, the lack of gene flow was evident in the networks analysis. However, especially in anemophilous tree species, gene flow (the product of effective population size and rate of migration among populations) can overtake the negative effects of fragmentation. Such a case is reflected in the Greek populations, which, due to wider extension and more continuous distribution compared to the Italian populations, exhibited a stronger isolation-by-distance along the latitudinal gradient extending toward the Balkan and the Alps.

Our study revealed the existence of historical gene flow between the populations from Southern Apennines and Balkan Peninsula, which is already reported for other tree species. Indeed, studying genetic differentiation and phylogeny of beech on the Balkans employing isoenzyme markers, Gömöry et al. (1999) and Magri et al. (2006) reported that despite nowadays geographical isolation, a similar genetic structure was observed between beech populations from Balkans and southern Italy. Pieces of evidence of common haplotypes, which grouped Italian and Balkan populations, were also found in Turkey oak (*Quercus cerris*; Bagnoli et al., 2016). Similarly, studies on silver fir (*Abies alba*) in the Apennines and Balkans found genetic similarity between the southern Apennines and eastern European populations (Belletti et al., 2016; Piotti et al., 2017). These studies are in agreement with ours and point toward the existence of historical gene flow between southern Italy and the Balkan peninsula (Carabeo et al., 2016), when the Apennine and Balkan Peninsulas were connected during Pleistocene at glaciation periods and concomitant sea-level drops (Nieto Feliner, 2014).

### Suggestions for Conservation

Populations along the Apennines in Italy represent examples of MaP populations, characterized by little extension and patchy distribution, low genetic diversity, low gene flow, and high differentiation. In Greece, populations are at the rear edge of the species continuous natural distribution do not show any evidence of genetic erosion. Hence, they represent a case where range dynamics, driven by past climate dynamics, played a major role in shaping the genetic structure. However, their rear edge location is a cause of concern about their preservation, since the rise of the isothermals and the drought spells extension forecasted for the Mediterranean region may endanger their gene pools. Since high levels of genetic variation are expected to increase the potential of the species to respond to selective pressure, we suggest that populations with higher genetic diversity and gene flow, such as the sites in Greece and also in the southern Apennines, can be considered for the establishment of *in situ* gene conservation units. Concurrently, populations showing peculiar gene pools are also considered as valuable source material for genetic conservation programs. In this regard, the stands of silver birch in the central Apennines and those at the lower elevations are genetically distinct and should also be targeted for conservation efforts, likely *ex situ*, as their long-term *in situ* adaptive potential may be compromised. However, genotypes able to survive and reproduce in warmer and drier conditions are expected to be selectively advantaged in local persistence and during migrations at higher latitudes and, particularly in the Mediterranean basin, altitudes. Under this perspective, with ongoing climate changes, these populations can be a source of genetic novelty for introducing and reinforcing standing genetic variation in other parts of the range.

## Conclusion

This study concentrates on the genetic structure of silver birch in the southern distribution range, focusing on the Italian and Greek marginal populations compared to central populations of the Alps and central Balkans. It has shown that it is not the peripherality (geographic location) or even the ecological marginality issue that may shape the genetic diversity and structure of MaP populations, but primarily their position as part of the continuous range or as disjunct populations. Greek populations, besides being at the edge of the natural distribution and in an ecologically marginal environment, retain the genetic characteristics typical of central populations due to their genetic connectivity with the rest of the natural continuous range. On the other hand, Italian MaP populations are clearly disjunct. Their genetic composition is the direct outcome of peripheral location and/or ecological marginality, compounded by disjunction and lack of gene flow. The results are important for the protection of the silver birch genetic resources in these areas. A future goal is to extend this study of putatively neutral genetic variation to an investigation of quantitative genetic variation in populations across the geographical range in order to gain a better understanding of the future adaptive potential of the southern and south-eastern silver birch populations.

## Data Availability Statement

The datasets generated for this study are available on request to the corresponding author.

## Author Contributions

GD, FD, and FA conceived and designed the study. GD, IG, EA, PB, and SS carried out fieldwork. AT processed samples, in collaboration with colleagues in CNR-IRET in Porano. GD, AT, and CM designed and performed the statistical analyses. GD wrote the original draft and all authors contributed in preparing the manuscript in its final form. All authors have read and approved the final manuscript.

## Conflict of Interest

The authors declare that the research was conducted in the absence of any commercial or financial relationships that could be construed as a potential conflict of interest.

## Footnotes

1 https://sites.ualberta.ca/∼ahamann/data.html

## References

[B1] Adamson K. R. Hughes P. D. Woodward J. C. (2013). Pleistocene glaciation of the Mediterranean mountains. *Quat. Newslett.* 131 2–15. 11918780

[B2] Allen R. Siegert M. J. Payne A. J. (2008). Reconstructing glacier-based climates of LGM Europe and Russia – Part 3: comparison with previous climate reconstructions. *Clim. Past* 4 265–280. 10.5194/cp-4-265-2008

[B3] Bagnoli F. Tsuda Y. Fineschi S. Bruschi P. Magri D. Zhelev P. (2016). Combining molecular and fossil data to infer demographic history of *Quercus cerris*: insights on European eastern glacial refugia. *J. Biogeogr.* 43 679–690. 10.1111/jbi.12673

[B4] Beck P. Caudullo G. de Rigo D. Tinner W. (2016). “*Betula pendula*, *Betula pubescens* and other birches in Europe: distribution, habitat, usage and threats,” in *European Atlas of Forest Tree Species*, eds San-Miguel-Ayanz J. de Rigo D. Caudullo G. Houston Durrant T. Mauri A., (Brussels: Publication Office of the European Union), e010226.

[B5] Belletti P. Ferrazzini D. Ducci F. De Rogatis A. Mucciarelli M. (2016). Genetic diversity of Italian populations of *Abies alba*. *Dendrobiology* 77 149–161. 10.12657/denbio.077.012

[B6] Birks H. J. B. Willis K. J. (2010). Alpines, trees, and refugia in Europe. *Plant Ecol. Divers.* 1 147–160. 10.1080/17550870802349146

[B7] Brown J. H. (1984). On the relationship between abundance and distribution of species. *Am. Nat.* 124 255–279. 10.1086/284267

[B8] Carabeo M. Simeone M. C. Cherubini M. Mattia C. Chiocchini F. Bertini L. (2016). Estimating the genetic diversity and structure of *Quercus trojana* Webb populations in Italy by SSRs: implications for management and conservation. *Can. J. For. Res.* 47 331–339. 10.1139/cjfr-2016-0311

[B9] Caudullo G. Welk E. San-Miguel-Ayanz J. (2017). Chorological maps for the main European woody species. *Data Brief* 12 662–666. 10.1016/j.dib 28560272 PMC5435575

[B10] Chapuis M.-P. Estoup A. (2007). Microsatellite null alleles and estimation of population differentiation. *Mol. Biol. Evol.* 24 621–631. 10.1093/molbev/msl191 17150975

[B11] Daly C. Halbleib M. Smith J. I. Gibson W. P. Doggett M. K. Taylor G. H. (2008). Physiographically sensitive mapping of climatological temperature and precipitation across the conterminous United States. *Int. J. Climatol.* 28 2031–2064. 10.1002/joc.1688

[B12] Do C. Waples R. S. Peel D. Macbeth G. M. Tillett B. J. Ovenden J. R. (2014). NeEstimator V2: re-implementation of software for the estimation of contemporary effective population size (Ne) from genetic data. *Mol. Ecol. Resour.* 14 209–214. 10.1111/1755-0998.12157 23992227

[B13] Earl D. A. vonHoldt B. M. (2012). STRUCTURE HARVESTER: a website and program for visualizing STRUCTURE output and implementing the Evanno method. *Conserv. Genet. Resourc.* 4 359–361. 10.1007/s12686-011-9548-7

[B14] Eckert C. G. Samis K. E. Lougheed S. C. (2008). Genetic variation across species’ geographical ranges: the central-marginal hypothesis and beyond. *Mol. Ecol.* 17 1170–1188. 10.1111/j.1365-294X.2007.03659.x 18302683

[B15] Evanno G. Regnaut S. Goudet J. (2005). Detecting the number of clusters of individuals using the software STRUCTURE: a simulation study. *Mol. Ecol.* 14 2611–2620. 10.1111/j.1365-294X.2005.02553.x 15969739

[B16] Excoffier L. Lischer H. E. L. (2010). Arlequin suite ver 3.5: A new series of programs to perform population genetics analyses under Linux and Windows. *Mol. Ecol. Resour.* 10 564–567. 10.1111/j.1755-0998.2010.02847.x 21565059

[B17] Fady B. Aravanopoulos F. A. Alizoti P. Mátyás C. von Wühlisch G. Westergren M. (2016). Evolution-based approach needed for the conservation and silviculture of peripheral forest tree populations. *For. Ecol. Manag.* 375 66–75. 10.1016/j.foreco.2016.05.015

[B18] Gömöry D. Paule L. Brus R. Zhelev P. Tomović Z. Gračan J. (1999). Genetic differentiation and phylogeny of beech on the Balkan peninsula. *J. Evol. Biol.* 12 746–754. 10.1046/j.1420-9101.1999.00076.x

[B19] Hampe A. Petit R. J. (2005). Conserving biodiversity under climate change: the rear edge matters. *Ecol. Lett.* 8 461–467. 10.1111/j.1461-0248.2005.00739.x 21352449

[B20] Harter D. E. Jentsch A. Durka W. (2015). Holocene re-colonisation, central-marginal distribution and habitat specialisation shape population genetic patterns within an Atlantic European grass species. *Plant Biol.* 17 684–693. 10.1111/plb.12269 25266560

[B21] Havrdová A. Douda J. Krak K. Vít P. Hadincová V. Zákravskı P. (2015). Higher genetic diversity in recolonized areas than in refugia of *Alnus glutinosa* triggered by continent-wide lineage admixture. *Mol. Ecol.* 24 4759–4777. 10.1111/mec.13348 26290117

[B22] Hewitt G. M. (2004). Genetic consequences of climatic oscillations in the quaternary. *Philos. Trans. R. Soc. Lond. B Biol. Sci.* 359 183–195. 10.1098/rstb.2003.1388 15101575 PMC1693318

[B23] IPCC (2013). Global climate projections. In Stocker T. F. Qin D. Plattner G. K. Tignor M. Allen S. K. Boschung J. Nauels A. Xia Y V. B Midgley P. (Eds.). *Climate change 2013: The physical science basis. Contribution of Working Group I to the Fifth Assessment Report of the Intergovernmental Panel on Climate Change.* Cambridge: Cambridge University Press, 867–952.

[B24] Jadwiszczak K. A. (2012). What can molecular markers tell us about the glacial and postglacial histories of European birches? *Silva. Fenn.* 46 733–745. 10.14214/sf.923

[B25] Jakobsson M. Rosenberg N. A. (2007). CLUMPP: a cluster matching and permutation program for dealing with label switching and multimodality in analysis of population structure. *Bioinformatics* 23 1801–1806. 10.1093/bioinformatics/btm233 17485429

[B26] Kalinowski S. T. (2005a). Do polymorphic loci require large sample sizes to estimate genetic distances? *Heredity* 94 33–36. 10.1038/sj.hdy.6800548 15329660

[B27] Kalinowski S. T. (2005b). HP-RARE 1.0: a computer program for performing rarefaction on measures of allelic richness. *Mol. Ecol. Notes* 5 187–189. 10.1111/j.1471-8286.2004.00845.x

[B28] Kivelä M. Arnaud-Haond S. Saramäki J. (2015). EDENetworks: a user-friendly software to build and analyse networks in biogeography, ecology and population genetics. *Mol. Ecol. Resour.* 15 117–122. 10.1111/1755-0998.12290 24902875

[B29] Kremer A. Potts B. M. Delzon S. (2014). Genetic divergence in forest trees: understanding the consequences of climate change. *Funct. Ecol.* 28 22–36. 10.1111/1365-2435.12169

[B30] Kulju K. K. M. Pekkinen M. Varvio S. (2004). Twenty-three microsatellite primer pairs for *Betula pendula* (Betulaceae). *Mol. Ecol. Notes.* 4 471–473. 10.1111/j.1471-8286.2004.00704.x

[B31] Leonardi S. Piovani P. Scalfi M. Piotti A. Giannini R. Menozzi P. (2012). Effect of habitat fragmentation on the genetic diversity and structure of peripheral populations of beech in central Italy. *J. Hered* 103 408–417. 10.1093/jhered/ess004 22496339

[B32] Luikart G. Cornuet J.-M. (1998). Empirical evaluation of a test for identifying recently bottlenecked populations from allele frequency data. *Conserv. Biol.* 12 228–237. 10.1111/j.1523-1739.1998.96388.x

[B33] Magri D. (2010). Persistence of tree taxa in Europe and Quaternary climate changes. *Quat. Int.* 219 145–151. 10.1016/j.quaint.2009.10.032

[B34] Magri D. Agrillo E. Di Rita F. Furlanetto G. Pini R. Ravazzi C. (2015). Holocene dynamics of tree taxa populations in Italy. *Rev. Palaeobot. Palynol.* 218 267–284. 10.1016/j.revpalbo.2014.08.012

[B35] Magri D. Vendramin G. G. Comps B. Dupanloup I. Geburek T. Gömöry D. (2006). A new scenario for the quaternary history of European beech populations: palaeobotanical evidence and genetic consequences. *New Phytol.* 171 199–221. 10.1111/j.1469-8137.2006.01740.x 16771995

[B36] Maliouchenko O. Palmé A. E. Buonamici A. Vendramin G. G. Lascoux M. (2007). Comparative phylogeography and population structure of European *Betula* species, with particular focus on *B. pendula* and *B. pubescens*. *J. Biogeogr.* 34 1601–1610. 10.1111/j.1365-2699.2007.01729.x

[B37] Nei M. (1973). Analysis of gene diversity in subdivided populations. *Proc. Natl. Acad. Sci. U.S.A.* 70 3321–3323. 10.1073/pnas.70.12.3321 4519626 PMC427228

[B38] Nieto Feliner G. (2014). Patterns and processes in plant phylogeography in the Mediterranean Basin. A review. *Perspect. Plant Ecol. Evol. Syst.* 16 265–278. 10.1016/j.ppees.2014.07.002

[B39] Nomura T. (2008). Estimation of effective number of breeders from molecular coancestry of single cohort sample. *Evol. Appl.* 1 462–474. 10.1111/j.1752-4571.2008.00015.x 25567728 PMC3352377

[B40] Normand S. Treier U. A. Vad Odgaard B. (2011). Tree refugia and slow forest development in response to post− LGM warming in North−Eastern European Russia. *Front. Biogeogr.* 2 91–93.

[B41] Ortego J. Riordan E. C. Gugger P. F. Sork V. L. (2012). Influence of environmental heterogeneity on genetic diversity and structure in an endemic southern Californian oak. *Mol. Ecol.* 21 3210–3223. 10.1111/j.1365-294x.2012.05591.x 22548448

[B42] Palmé A. E. Su Q. Rautenberg A. Manni F. Lascoux M. (2003). Postglacial recolonization and cpDNA variation of silver birch. *Betula. Pendula*. *Mol. Ecol.* 12 201–212. 10.1046/j.1365-294X.2003.01724.x 12492888

[B43] Peakall R. Smouse P. E. (2006). GENALEX 6: genetic analysis in Excel. Population genetic software for teaching and research. *Mol. Ecol.* 6 288–295. 10.1111/j.1471-8286.2005.01155.x PMC3463245 22820204

[B44] Peakall R. Smouse P. E. (2012). GenAlEx 6.5: genetic analysis in Excel. Population genetic software for teaching and research-an update. *Bioinformatics* 28 2537–2539. 10.1093/bioinformatics/bts460 22820204 PMC3463245

[B45] Petit R. Aguinagalde I. de Beaulieu J. L. Bittkau C. Brewer S. Cheddadi R. (2003). Glacial refugia: hotspots but not melting pots of genetic diversity. *Science* 300 1563–1565. 10.1126/science.1083264 12791991

[B46] Piotti A. Leonarduzzi C. Postolache D. Bagnoli F. Spanu I. Brousseau L. (2017). Unexpected scenarios from Mediterranean refugial areas: disentangling complex demographic dynamics along the Apennine distribution of silver fir. *J. Biogeogr.* 44 1547–1558. 10.1111/jbi.13011

[B47] Piry S. Luikart G. Cornuet J.-M. (1999). BOTTLENECK: a computer program for detecting recent reductions in the effective population size using allele frequency data. *J. Hered.* 90 502–503. 10.1093/jhered/90.4.502

[B48] Pritchard J. K. Stephens M. Donnelly P. (2000). Inference of population structure using multilocus genotype data. *Genetics* 155 945–959. 10835412 10.1093/genetics/155.2.945 PMC1461096

[B49] R Core Team (2019). *R: A Language and Environment for Statistical Computing.* Vienna: R Foundation for Statistical Computing.

[B50] Ravenscroft C. H. Whitlock R. Fridley J. D. (2015). Rapid genetic divergence in response to 15 years of simulated climate change. *Glob. Change Biol.* 21 4165–4176. 10.1111/gcb.12966 26311135 PMC4975715

[B51] Rosenberg N. A. (2004). DISTRUCT: a program for the graphical display of population structure. *Mol. Ecol. Notes* 4 137–138. 10.1046/j.1471-8286.2003.00566.x

[B52] R Studio Team (2016). *RStudio: Integrated Development Environment for R.* Boston, MA: RStudio, Inc.

[B53] Ryman N. Palm S. (2006). POWSIM: a computer program for assessing statistical power when testing for genetic differentiation. *Mol. Ecol. Notes* 6 600–602. 10.1111/j.1471-8286.2006.01378.x 11703649

[B54] Salojärvi J. Smolander O.-P. Nieminen K. Rajaraman S. Safronov O. Safdari P. (2017). Genome sequencing and population genomic analyses provide insights into the adaptive landscape of silver birch. *Nat. Genet.* 49 904–912. 10.1038/ng.3862 28481341

[B55] Schierenbeck K. A. (2017). Population-level genetic variation and climate change in a biodiversity hotspot. *Ann. Bot.* 119 215–228. 10.1093/aob/mcw214 28069633 PMC5321061

[B56] Schoettle A. W. Goodrich B. A. Hipkins V. Richards C. Kray J. (2012). Geographic patterns of genetic variation and population structure in *Pinus aristata*, Rocky Mountain bristlecone pine. *Can. J. For. Res.* 42 23–37. 10.1139/x11-152

[B57] Sexton J. P. McIntyre P. J. Angert A. L. Rice K. J. (2009). Evolution and ecology of species range limits. *Annu. Rev. Ecol. Evol. Syst.* 40 415–436. 10.1146/annurev.ecolsys.110308.120317

[B58] Stewart J. R. Lister A. M. Barnes I. Dalén L. (2010). Refugia revisited: individualistic responses of species in space and time. *Proc. R. Soc. B* 277 661–671. 10.1098/rspb.2009.1272 19864280 PMC2842738

[B59] Svenning J.-C. Normand S. Kageyama M. (2008). Glacial refugia of temperate trees in Europe: insights from species distribution modelling. *J. Ecol.* 96 1117–1127. 10.1111/j.1365-2745.2008.01422.x

[B60] Tsuda Y. Semerikov V. Sebastiani F. Vendramin G. G. Lascoux M. (2017). Multispecies genetic structure and hybridization in the genus *Betula* across Eurasia. *Mol. Ecol.* 26 589–605. 10.1111/mec.13885 27763698

[B61] Tzedakis P. C. Emerson B. C. Hewitt G. M. (2013). Cryptic or mystic? Glacial tree refugia in northern Europe. *Trends Ecol. Evol.* 28 696–704. 10.1016/j.tree.2013.09.001 24091207

[B62] Vakkari P. (2009). *EUFORGEN Technical Guidelines for Genetic Conservation and Use of Silver Birch (Betula pendula)*. Rome: Bioversity International, 6 Available online at: http://www.euforgen.org/publications/publication/betula-pendula-technical-guidelines-for-genetic-conservation-and-use-for-silver-birch/

[B63] Väliranta M. Kaakinen A. Kuhry P. Kultti S. Salonen J. S. Seppä H. (2011). Scattered late-glacial and early Holocene tree populations as dispersal nuclei for forest development in north-eastern European Russia. *J. Biogeogr.* 38 922–932. 10.1111/j.1365-2699.2010.02448.x

[B64] Waples R. S. Do C. (2008). LDNE: a program for estimating effective population size from data on linkage disequilibrium. *Mol. Ecol. Resour.* 8 753–756. 10.1111/j.1755-0998.2007.02061.x 21585883

[B65] Willis K. J. van Andel T. H. (2004). The environments of central and eastern Europe during the last Glaciation. *Quat. Sci. Rev.* 23 2368–2387. 10.1016/j.quascirev.2004.06.002 18665223

[B66] Yousefzadeh H. Hosseinzadeh Colagar A. Fallah F. (2016). Genetic diversity of geographically isolated Iranian populations of *Betula pendula* roth: implications for conservation. *Silva. Fenn.* 50:1516 10.14214/sf.1516

[B67] Zhdanova O. L. Pudovkin A. I. (2008). Nb_HetEx: a program to estimate the effective number of breeders. *J. Hered.* 99 694–695. 10.1093/jhered/esn061 18703539

