# Supplementary material for: Genetic Analysis by nuSSR Markers of Silver Birch (Betula pendula Roth) Populations in Their Southern European Distribution Range
Source: Front Plant Sci. 2020 Mar 24;11:310. doi: 10.3389/fpls.2020.00310 (PMC7108150; doi:10.3389/fpls.2020.00310)
Supplement: Supplementary file 1 [file Data_Sheet_1.docx]

Supplementary Material

# Supplementary Figures and Tables

## Supplementary Tables

**Supplementary Table S1.** List of the 15 climatic variables used by the ClimateEU software

| **Annual variables** |  |
| --- | --- |
| *Directly calculated* |  |
| MAT | mean annual temperature (°C), |
| TD | temperature difference between MWMT and MCMT, or continentality (°C) |
| MAP | mean annual precipitation (mm), |
| MSP | mean summer (May to Sept.) precipitation (mm), |
| AHM | annual heat:moisture index (MAT+10)/(MAP/1000)) |
| SHM | summer heat:moisture index ((MWMT)/(MSP/1000)) |
| *Derived* |  |
| DD<0 | degree-days below 0 °C, chilling degree-days |
| DD>5 | degree-days above 5 °C, growing degree-days |
| DD<18 | degree-days below 18 °C, heating degree-days |
| DD>18 | degree-days above 18 °C, cooling degree-days |
| NFFD | the number of frost-free days |
| bFFP | the Julian date on which FFP begins |
| eFFP | the Julian date on which FFP ends |
| FFP | frost-free period |
| CMD | Hargreaves climatic moisture deficit |

## Supplementary Figures

**Supplementary Figure S1.** Second order of change of the log-likelihood of the data (ΔK) as a function of K, calculated over 20 replicates, associated with the results shown in Figure 3.

**Supplementary Figure S2.** (a) Principal Component Analysis of the 14 sites and (b) loading factors of the 15 variables.
